# Supplementary material for: The Management of HIV Care Services in Central and Eastern Europe: Data from the Euroguidelines in Central and Eastern Europe Network Group
Source: Int J Environ Res Public Health. 2022 Jun 21;19(13):7595. doi: 10.3390/ijerph19137595 (PMC9265352; doi:10.3390/ijerph19137595)
Supplement: Supplementary file 1 [file ijerph-19-07595-s001.zip › ijerph-1736388-supplementary.pdf]

**Questionnaire – models of HIV care during COVID-19 times**

The achievement of global 90-90-90 targets in HIV testing, prevention and treatment was severely threatened by the COVID-19 pandemic. In this context, there was a need for new models of HIV care.
In this questionnaire we are asking what new strategies of HIV care were implemented during challenging COVID-19 time in Central and Eastern Europe Countries and answers for the relevant questions – how we improved service for key populations and how we can adapt the lessons learned to the post-COVID-19 period.

**Telehealth** - the provision of healthcare remotely by means of telecommunications technology

**PERSONAL DATA**

1. Full name and surname…………………….
2. E-mail………..
3. Country:………………..
4. Clinic full name (affiliation):………………………………..
5. Medical speciality: …………………………
    a. ID physician (completed or in curse)
    b. Other specialty physician
    c. Other medical personnel
    d. Other non-medical personnel

**GENERAL INFORMATION**

6. Please describe the organisation of your centre:
    a. based on out-patient clinics
    b. ID hospital plus out-patients clinic
    c. general hospital plus out-patient clinic
    d. other (please specify)
7. Are you directly involved in HIV care:
    a. Yes
    b. No
    c. Other:…………………………
8. Is your country in a lock-down situation at the moment?
    a. Yes
    b. No

   c. No, but a lock-down is likely soon
   d. No, we have completed lock-down
9. Was your center affected by the COVID-19 outbreak?
   a. Yes
   b. no
10. How is your HIV clinic operating now:
   d. Normally
   e. Shorter hours but admitting
   f. Less personnel but admitting
   g. Normal activity suspended
   h. HIV clinic is closed
   i. We combine face-to face visits and telehealth
   j. Other: …………………………

**TESTING**

11. Are people in your country able to access testing for HIV?
   a. Yes
   b. No

12. Was there any decline in testing for HIV after March 2020?
   a. Yes
   b. No
   c. I don't know
13. If yes, what were the reasons? (multiple answer possible)
   a. testing facilities were closed
   b. people were unable to travel to health facilities due to travel restrictions
   c. people were avoiding going to testing facilities due to COVID-19
   d. the lack of staff
   e. other
14. Were there any new testing for HIV methods introduced after March 2020?
   a. Rapid self tests
   b. Mobile testing points
   c. Other (please specify)
   d. No

**NEW MODELS OF CARE**

15. Were there any telehealth tools used **before** COVID-19 pandemic as a routine?
    a. Yes
    b. No
16. If yes, what telehealth tools were used **before** COVID-19 pandemic as a routine in your center?
    a. phone
    b. e-mail
    c. other messaging services
    d. virtual clinics, videocalls (eg. via Skype)
    e. on-line health parameters assessment (eg. ECG)
    f. dedicated smartphone apps
    g. other (please specify)
17. Which **new** services or tools (if any) were introduced after March 2020?
    a. Telehealth (the provision of healthcare remotely by means of telecommunications technology.)
    b. home-based HIV testing
    c. longer period of time in drug supplies
    d. Medication delivery programmes
    e. Community ART distribution points
    f. Home visits
    g. Mobile health centres
    h. None of the above
    i. Other (please specify)
18. For how long were you supplying ARVs for your patients **before** March 2020:
    a. 2 weeks
    b. 1 month
    c. 2-3 months
    d. 4-6 months
19. For how long were you supplying ARVs for your patients **after** March 2020 **by** May 2021?
    a. 2 weeks
    b. 1 month
    c. 2-3 months
    d. 4-6 months
    e. More
20. For how long are you supplying ARVs for your patients now?

a. 2 weeks
b. 1 month
c. 2-3 months
d. 4-6 months
e. More

21. How often had you measured the viral load in your patients **before** March 2020?
    a. Every 1 month
    b. Every 3 months
    c. Every 6 months
    d. Other
22. How often had you measured the viral load in your patients **after** March 2020 **by** May 2021?
    a. Every 1 month
    b. Every 3 months
    c. Every 6 months
    d. Other

23. How often do you measure the viral load in your patients now?
    a. Every 1 month
    b. Every 3 months
    c. Every 6 months
    d. Other
24. How much did face-to face visits declined **after March 2020 by the end of 2020** (in compare to the times before COVID-19 pandemic)
    a. 0%,
    b. 0-25%,
    c. 25-50%
    d. >50%
25. How much did face-to face visits declined **from the end of 2021 by the end of May 2021** (in compare to the times before COVID-19 pandemic)
    a. 0%,
    b. 0-25%,
    c. 25-50%
    d. >50%

**KEY POPULATIONS**

26. Did you have lower access to specific group of patients during COVID-19 pandemic:
    a. Yes
    b. No
27. If yes, which group it was related to:
    a. Migrants
    b. IVDU
    c. Chemsex users
    d. People in prisons
    e. Other (please specify)
28. If yes, what was the probable reason?related to current pandemic situation (eg. Social worker or translator not available, mobile clinics not operating): …………….
29. Was there any tools in your center to take care about above patients?
    a. Please make a comment

30. Any other comments (up to 50 words): ……………………………………..
